# Six Months of Bikram Yoga: Longitudinal Effects on Body Fat Reduction and Age-Related Responses in Adult Women

**DOI:** 10.3390/healthcare14081032

**Published:** 2026-04-14

**Authors:** Federico Zoila, Daniela Cagnetta, Sergio Bellantonio, Pasquale Simeone, Paola Lanuti, Maria Antonietta Panaro, Laura Civita, Laura Antonucci, Chiara Porro

**Affiliations:** 1 Department of Clinical and Experimental Medicine, University of Foggia, I-71100 Foggia, Italy; daniela.cagnetta.dc@gmail.com (D.C.); pasquale.simeone@unifg.it (P.S.); laura.antonucci@unifg.it (L.A.); 2 Department of Neurosciences, Biomedicine and Movement Sciences, University of Verona, I-37134 Verona, Italy; 3 Department of Social Sciences, University of Foggia, I-71100 Foggia, Italy; sergio.bellantonio@unifg.it; 4 Department of Medicine and Aging Sciences, Center for Advanced Studies and Technology-CAST, University G. d’Annunzio of Chieti-Pescara, I-66100 Chieti, Italy; paola.lanuti@unich.it; 5 Flow Cytometry Core Facility, Center for Advanced Studies and Technology-CAST, University G. d’Annunzio of Chieti-Pescara, I-66100 Chieti, Italy; 6 Department of Biosciences, Biotechnologies and Environment, University of Bari, I-70121 Bari, Italy; mariaantonietta.panaro@uniba.it; 7 Socio-Health District No.3, ASL Bari, I-70032 Bitonto, Italy; laura.civita@asl.bari.it

**Keywords:** Bikram yoga, hot yoga, body fat, adult women, exercise intervention, obesity, metabolic syndrome, active aging

## Abstract

**Background:** Bikram yoga, a form of hot yoga practiced in heated environments, has been associated with improvements in flexibility, body composition, and overall well-being. However, longitudinal evidence on its effects in adult women remains limited. Obesity/metabolic syndrome (MetS) is highly prevalent among adult women worldwide, with estimates exceeding 40% in middle-aged populations, underscoring the need for low-impact interventions targeting adiposity and age-related metabolic risks. This study evaluated the effects of 6-month Bikram yoga on body fat percentage (%BF) in adult women, with age-stratified analyses. **Methods:** Twenty-two women (20–65 years) participated in a structured Bikram yoga program consisting of three weekly sessions (90 min, 26 postures + 2 breathing exercises, 40 °C, 40% humidity) over six months. Anthropometric assessments (8 skinfolds, 5 body circumferences, weight, and height) were conducted at T0, T1 (~45 days), T2 (~90 days), and T3 (6 months). %BF was estimated using multiple validated prediction equations integrated into the Exercise Science Toolkit. **Results:** A significant and progressive reduction in %BF was observed across the sample: −3.71% at T1 (*p* < 0.0001) and −6.07 at T3 (*p* < 0.0001) compared to the baseline. Positive outcomes were consistent across all age subgroups: subgroup A (20–35 years, T3 −6.62%), subgroup B (36–50 years, T3 −5.96%), and subgroup C (51–65 years, T3 −5.39%). Decreased inter-subject variability (SD) suggests a similar direction of change among participants. **Conclusions:** Regular Bikram yoga practice (three sessions per week for six months) was associated with significantly and consistently reduced %BF among adult women aged 20–65, exceeding the clinical threshold (>5%) for metabolic benefits. Effects were evident after six weeks and remained across all age subgroups, suggesting that Bikram yoga may represent an effective, low-impact intervention for health promotion and active aging.

## 1. Introduction

Overweight and obesity represent a major public health challenge, particularly among adult women, due to their association with cardiometabolic risk and long-term health complications. Lifestyle interventions that combine physical activity and behavioral changes remain the cornerstone of obesity management, although adherence to traditional exercise programs is limited [1]. In recent years, yoga has emerged as a promising mind–body intervention that combines physical activity, breathing techniques, and behavioral components. Growing evidence suggests that yoga may positively influence body composition, cardiovascular risk factors, and lifestyle behaviors in populations with overweight or obesity [2]. This discipline constitutes a multifaceted system characterized by numerous variations that prioritize different physical, mental, and spiritual dimensions, rather than a single, monolithic practice. Over time, yoga has evolved into multiple schools and styles, each with distinct practices and underlying philosophies, making it suitable for diverse populations with varying needs and preferences [3].

In Western cultures, yoga and meditation have become popular for reducing stress, enhancing well-being, and promoting overall health [4]. Recently, yoga has been the subject of research as a therapeutic measure to prevent or treat medical conditions such as stress, insomnia, obesity, anxiety, diabetes, hypertension, oxidative stress, glucose tolerance, dyslipidemia [5]. The management of body composition is a cornerstone of global public health. Recent data indicate that over 40% of adult women worldwide are overweight or affected by obesity and chronic conditions intrinsically linked to an increased risk of type 2 diabetes, hypertension, and metabolic syndrome [6]. In adult women, age-related hormonal shifts often exacerbate visceral fat accumulation and lean mass decline, accelerating metabolic dysfunction. Given these trends, there is an urgent need for sustainable, low-impact exercise interventions that can effectively manage adiposity-related risks throughout the female lifespan [7]. Engagement in yoga postures (asanas) and breathing techniques (pranayama) has been shown to contribute to the regulation of total serum cholesterol, low-density lipoprotein (LDL), very-low-density lipoprotein (VLDL), and triglyceride levels [5]. Furthermore, multiple studies have documented the beneficial effects of yoga in metabolic disorders, including diabetes and metabolic syndrome [8].

In recent years, the practice of performing yoga within heated environments has become increasingly popular, with proposed benefits including increased flexibility, general fitness, reduced systemic inflammation, improved body composition, reduced blood pressure, increased muscular strength, and improvements in multiple psychological domains, including mood and anxiety-related parameters [9,10]. This practice is called hot yoga, and typically, sessions are conducted at ambient temperatures of 32–41 °C (90–105° F), with relative humidity levels between 40%and 70%, contingent upon the specific style of practice [11]. Bikram yoga is a specific system of hatha yoga that incorporates a 90-minute, unchanging sequence of 26 hatha yoga asanas (postures) combined with two breathing exercises performed in a heated environment (40.6 °C, 40% humidity) and instructional dialogue [12,13]. Research studies investigating the health benefits associated with this practice have been emerging in recent years [14]. Studies have demonstrated that eight weeks of Bikram yoga practice can enhance health outcomes in apparently healthy adults and those at risk for chronic disease [15]. Bikram yoga consists of beginner-level asanas, making it suitable for virtually all fitness levels. However, despite its applicability and potential benefits, challenges remain in successfully applying this intervention in sedentary adults [16].

Bikram yoga has been investigated for a range of physiological and psychological outcomes. Consistent evidence indicates improvements in musculoskeletal performance, particularly strength, flexibility, and balance [17]. Effects on body composition, bone health, vascular, and metabolic function are more variable, with some studies reporting modest reductions in adiposity, preservation of bone mineral density, and improvements in endothelial function and glucose tolerance [9,14,18]. Recent evidence has further highlighted the potential role of hot yoga in improving body composition and metabolic health. Beyond physical outcomes, yoga has been widely studied for its psychological and neurological benefits, including applications in neurodegenerative disorders and mental health. Psychological outcomes, including reductions in perceived stress and enhanced mindfulness, have also been observed, and emerging trials suggest potential benefits for depressive symptoms and affect regulation. These multisystem effects highlight the broad therapeutic potential of yoga as a holistic intervention [19,20]. The heated environment introduces additional cardiovascular and thermal stress, which may augment cellular and vascular adaptations. However, several studies indicate that benefits can also be achieved under thermoneutral conditions [21]. While these findings highlight the potential of Bikram yoga as a complementary health intervention, the current evidence base is constrained by small sample sizes, heterogeneous methodologies, and limited randomized controlled trials, particularly in clinical populations [22].

Obesity and MetS represent a global public health crisis, affecting over 40% of adult women and increasing risks for type 2 diabetes, hypertension, and cardiovascular disease through visceral fat accumulation, insulin resistance, and chronic low-grade inflammation [23]. Age-related hormonal changes in women exacerbate these issues, promoting sarcopenic obesity, a condition of concurrent muscle loss and adiposity that impairs metabolic health. Innovative exercise-based interventions, such as heated yoga practices like Bikram yoga, offer promising non-pharmacological strategies by enhancing fat oxidation, improving insulin sensitivity, and modulating adipokines (e.g., leptin and adiponectin) in overweight/obese populations [24]. Sorout et al. demonstrated that yoga interventions reduce metabolic risk factors, including waist circumference, triglycerides, and fasting glucose while improving quality of life in MetS patients [25].

Accurate body composition assessment is fundamental in exercise and health sciences; criterion techniques, including dual-energy X-ray absorptiometry (DXA) and hydrodensitometry, are frequently constrained by substantial financial costs, specialized technical requirements, and limited access in many clinical and research settings. In contrast, anthropometric approaches based on skinfolds and circumferences provide a cost-effective, portable, and broadly applicable alternative that can be implemented in a wide range of research, clinical, and field settings [26,27]. For these reasons, the present study employed validated anthropometric prediction equations integrated into the Exercise Science Toolkit (University of South Australia) to estimate %BF. This approach was chosen to maximize methodological feasibility and applicability across diverse contexts while maintaining acceptable levels of validity and reliability [28].

Building on this methodological framework, the present study aims to evaluate the longitudinal changes in body composition generated by a 6-month Bikram yoga program in a cohort of adult women. Specifically, we sought to analyze variations in %BF across four time points (T0, T1, T2, T3) in relation to participant age. We hypothesized that the intervention would produce a progressive reduction in adiposity and that the extent of these adjustments would be modulated by cumulative duration of practice, observing possible differences between age groups.

## 2. Materials and Methods

### 2.1. Study Design

**Figure 1:**
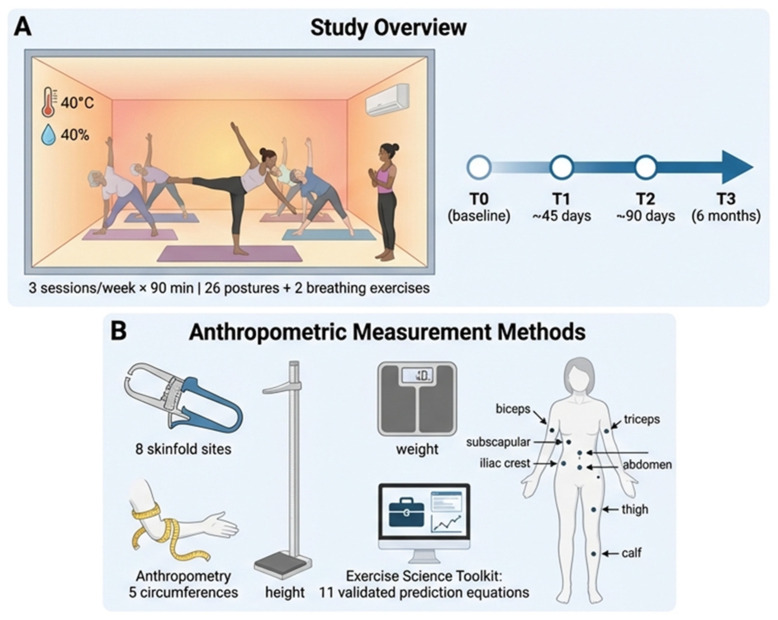
Study design and anthropometric assessment methods. (**A**) Overview of the 6-month Bikram yoga intervention, including environmental conditions (40 °C, 40% humidity), session frequency (3 sessions/week, 90 min), and the four assessment time points: baseline (T0), ~45 days (T1), ~90 days (T2), and 6 months (T3). (**B**) Anthropometric measurement procedures, including body mass, stature, eight skinfold sites, five circumferences, and %BF estimation using 11 validated prediction equations integrated into the Exercise Science Toolkit.

The study followed a prospective, longitudinal, single-arm observational design with repeated measurements over time. The participants were monitored for a six-month period, during which four anthropometric measurement sessions were conducted: T0 (baseline); T1 (approximately 45 days); T2 (approximately 90 days); and T3 (at the end of the six months). The aim was to evaluate progressive changes in body composition associated with Bikram yoga practice, with analyses stratified by age group. No control group was included. Figure 1 illustrates the Bikram yoga intervention and longitudinal study design.

### 2.2. Participants

Twenty-two voluntary women participants, aged between 20 and 65, were recruited through convenience sampling from a yoga center specializing in Bikram yoga. The inclusion criteria required participants aged between 20–65; to be beginners, defined as having practiced Bikram yoga for a maximum of a few weeks prior to the study. All participants were screened for medical eligibility and deemed fit to practice high-temperature yoga. Data collection was conducted in person, in accordance with applicable hygiene and safety regulations. Participants were instructed to maintain their usual dietary habits throughout the study period; however, no formal dietary monitoring or assessment was conducted. For the analysis of results, participants were also stratified into age subgroups: subgroup A (20–35 years), subgroup B (36–50 years), and subgroup C (51–65 years). This stratification allowed for the observation of individual and collective differences in the evolution of anthropometric parameters. The study was conducted in accordance with the Declaration of Helsinki, and the protocol was approved by the Ethics Committee of the Area 1 Inter-Provincial (protocol code Prot. n. 17/Seg. CE/2021, approved on 25 June 2021). All participants provided written informed consent before participation.

### 2.3. Intervention: Bikram Yoga Program

The study participants attended structured Bikram yoga classes. Each session lasted 90 min, divided into a fixed sequence of 26 postures (Asana), 2 breathing exercises (Pranayama), and was conducted in a heated room at approximately 40 °C and 40% relative humidity. The program lasted 6 months, with three sessions per week. The total number of sessions planned was approximately 72 per participant. Adherence was not formally monitored. However, participants were encouraged to attend all sessions, and no major issues with participation were informally reported during the intervention.

### 2.4. Anthropometric Measurements

The measurements were carried out four times: T0, T1, T2, and T3. Each participant was measured under standardized conditions, preferably in the morning, after rest, and in light clothing. The tools used were the Harpenden caliper (accuracy: ±1 mm, constant pressure of 10 g/mm^2^), anthropometry tape (precision to the millimeter), calibrated digital scale (precision ±100 g), and stadiometer (only at T0). The same operator performed all measurements to guarantee standardization and technical coherence. No formal intra-observer reliability assessment was conducted.

Anthropometric assessments were conducted according to the International Society for the Advancement of Kinanthropometry (ISAK) guidelines. Eight skinfolds (biceps, triceps, subcapular, supraspinale, abdominal, iliac crest, front thigh, and medial calf), five body circumferences (arm-relaxed, arm-flexed and tensed, waist-minimum, gluteal-hips, and calf-maximum), and other parameters (body mass, stature) were measured using standard procedures.

### 2.5. Data Analysis

The collected data were analyzed using the Exercise Science Toolkit (University of South Australia), which integrates validated prediction equations to estimate body density and %BF. Specifically, 11 equations were applied [29,30,31,32,33,34,35,36,37,38,39]. The software generated %BF estimates for each equation, and the final %BF value used for analysis was calculated as the mean of these estimates. This approach reduces reliance on a single equation and accounts for variability among population-specific prediction models. The data were then stored in Microsoft Excel, and a dataset was prepared including participants, measurement time points, and all anthropometric variables. Data are presented as the mean ± standard deviation (SD). To evaluate changes in %BF over the six-month intervention, a repeated measures ANOVA was performed in GraphPad Prism 8.0.2, with measurement time points (T0, T1, T2, T3) as the within-subject factor. When the ANOVA indicated a significant effect of time, post hoc comparisons were conducted using the Šidák correction. For the repeated measures ANOVA analyses, estimates of effect sizes are given in terms of partial eta-squared measures (*η_p_*^2^). The same statistical procedure was applied to the total sample and to age-based subgroups. For all analyses, statistical significance was set at *p* < 0.05.

## 3. Results

The study monitored the evolution of body composition in a heterogeneous group of 22 women, aged between 20 and 65, participating in a 6-month Bikram yoga program. Assessments were performed at four time points (T0, T1, T2, and T3) approximately 40–45 days apart. The outcome considered was %BF, estimated from skinfold measurements performed by the same operator. The study participants showed a statistically significant progressive reduction in %BF across the observation period.

**Figure 2:**
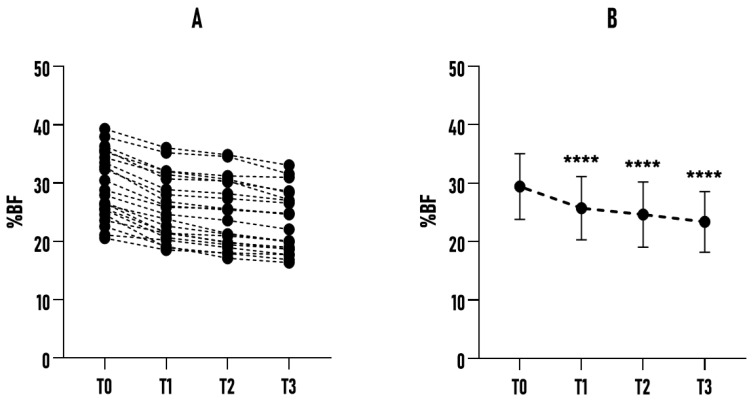
Effect of a 6-month Bikram yoga program on %BF in all participants (n = 22). (**A**) Individual changes in %BF across all participants at baseline (T0) and subsequent time points (T1, T2, T3). (**B**) Mean %BF values of the sample at each time point, presented with error bars (±SD). **** *p* < 0.0001 compared to the baseline.

As shown in Figure 2, the study participants’ sample exhibited a significant reduction of 3.71% (*p* < 0.0001) at T1 compared to the baseline (T0). This trend was confirmed at subsequent time points, with further declines resulting in an overall reduction of 6.07% between T0 and T3 (*p* < 0.0001; *η_p_*^2^ = 0.924). Another significant finding concerns the reduction in SD, which decreased from ±5.63% at T0 to ±5.20% at T3. This reduction in variability suggests a relatively consistent response to the protocol. These results indicate that Bikram yoga was associated with consistent reductions in %BF across the sample, showing a positive trend regardless of age, initial body composition, or fitness level, with no major outliers (ROUT method, GraphPad Prism 8.0.2). No increases in %BF were observed during follow-up. Bikram yoga, practiced three times a week, was associated with a systematic favorable impact on body composition, with a reduction in %BF evident within the first 45 days of practice. To understand age-related differences in body composition, particularly in %BF, the sample was stratified into three subgroups: A (20–35 years), B (36–50 years), and C (51–65 years). Even with this subdivision, the emerging data were particularly significant and showed consistent trends across the groups, albeit with some differences in intensity and variability.

**Figure 3:**
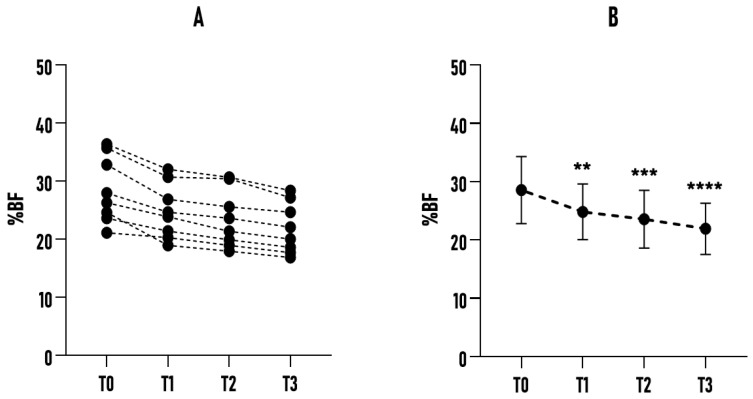
Effect of a 6-month Bikram yoga program on %BF in subgroup A (20–35 yrs, n = 8). (**A**) Individual changes in %BF across all subgroup participants at baseline (T0) and subsequent time points (T1, T2, T3). (**B**) Mean %BF values of the sample at each time point, presented with error bars (±SD). ** *p* < 0.01 compared to the baseline. *** *p* < 0.001 compared to the baseline. **** *p* < 0.0001 compared to the baseline.

In subgroup A (n = 8), a constant and marked reduction in %BF was observed over the six months (see Figure 3). The %BF decreased from an initial mean of 28.54% ± 5.75% at T0 to 21.92% ± 4.38% at T3 (*p* < 0.0001; *η_p_*^2^ = 0.911). The overall decrease was 6.62%, with a progressive reduction in intra-subject variability (as reflected by the lower SD), suggesting adaptation to the practice protocol. This subgroup showed a particularly efficient response to the Bikram yoga stimulus, possibly reflecting age-related differences in metabolic and hormonal reactivity. The consistent trend over time, with an average reduction of more than 2 percentage points at each measurement interval, confirms a favorable physiological response.

**Figure 4:**
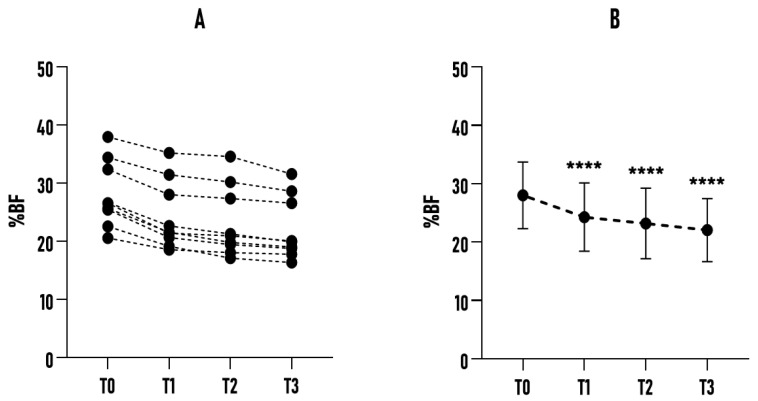
Effect of a 6-month Bikram yoga program on %BF in subgroup B (36–50 yrs. n = 9). (**A**) Individual changes in %BF across all subgroup participants at baseline (T0) and subsequent time points (T1, T2, T3). (**B**) Mean %BF values of the sample at each time point, presented with error bars (± SD). **** *p* < 0.0001 compared to the baseline.

In subgroup B (n = 9), the results were also positive and aligned with the overall trend, albeit with higher mean baseline values and standard deviations. At T0, mean %BF was 28.02% ± 5.70%, which progressively decreased to 22.06% ± 5.40% at T3. The overall reduction was approximately 5.96% (*p* < 0.0001; *η_p_*^2^ = 0.939). While maintaining an improving trend, this subgroup showed greater intra-subject variability (with a higher SD than the other groups), possibly reflecting more pronounced differences in lifestyle, metabolism, baseline status, and program adherence. Nevertheless, a statistically significant reduction was observed across successive time points (see Figure 4).

**Figure 5:**
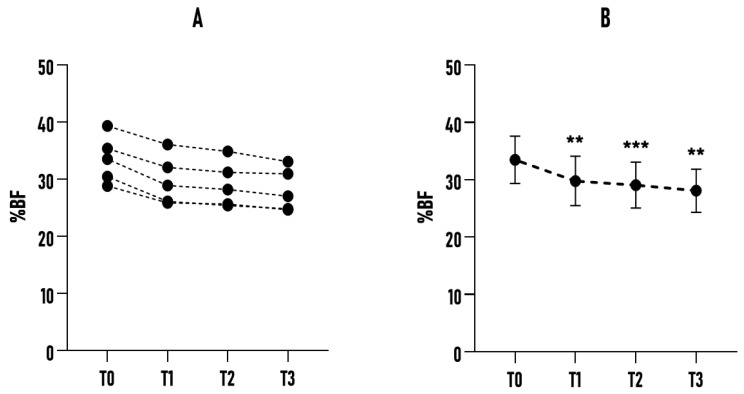
Effect of a 6-month Bikram yoga program on %BF in subgroup C (51–65 yrs n = 5). (**A**) Individual changes in %BF across all subgroup participants at baseline (T0) and subsequent time points (T1, T2, T3). (**B**) Mean %BF values of the sample at each time point, presented with error bars (± SD). ** *p* < 0.01 compared to the baseline. *** *p* < 0.001 compared to the baseline.

The subgroup C (n = 5) also showed positive results, with a favorable evolution of the anthropometric profile, although slightly more gradual than the other subgroups. At T0, the initial mean %BF was 33.48% ± 4.14%, while at T3 it was 28.09% ± 3.76%, resulting in a total reduction of approximately 5.39% (*p* < 0.01; *η_p_*^2^ = 0.960). Despite the higher baseline level, the reduction in %BF was evident and consistent over time, demonstrating that regular Bikram yoga practice can also be associated with beneficial effects in older individuals. The relatively low standard deviation across time points suggests limited inter-individual variability in the observed changes, which is an interesting finding despite the greater physiological heterogeneity typical of this age subgroup (see Figure 5).

When comparing the three subgroups, all age groups benefited from regular Bikram yoga practice, with a significant reduction in %BF. Subgroup A showed marked and consistent improvement; subgroup B had a high average response but greater variability; and subgroup C achieved solid, consistent results. Overall, these data (see Table 1) support the effectiveness of Bikram yoga in improving body composition across age groups, despite differences in response rates and individual variability. This suggests that Bikram yoga practice may be effectively adaptable to different target populations, making it a safe and beneficial physical activity for prevention and active aging. The analysis highlights that regular Bikram yoga practice (three sessions per week) may be an important factor in reducing %BF. Furthermore, younger participants tended to respond faster, while effectiveness remained stable in older participants.

No adverse events were reported during the intervention period. In particular, no participants experienced dizziness, dehydration, or symptoms related to heat intolerance.

## 4. Discussion

The study demonstrated a significant and progressive reduction in %BF over six months of regular Bikram yoga practice in adult women. The decrease was observed in the overall sample and across all age subgroups, indicating a consistent longitudinal trend. Overall, in a sample of 22 women aged 20–65, a consistent reduction in %BF was observed from T0 to T3, regardless of age. %BF showed a progressive decrease over the six-month intervention, suggesting that, if performed consistently, it may produce appreciable physiological improvements.

From a demographic perspective, an age-related pattern was observed: the 20–35 age group showed the most significant results, followed by the 36–50 and 51–65 age groups. While highlighting a lower responsiveness in older subjects, this trend demonstrates that Bikram yoga can be practiced beneficially even at a mature age, representing an effective form of exercise with low joint impact yet potentially high metabolic value.

The mean reduction of 6.07% in %BF observed in this study may have meaningful clinical relevance. Although some meta-analyses report mixed results regarding yoga’s impact on body fat percentage, with no significant changes found in several studies, others indicate modest improvements in anthropometric measures such as waist circumference and body weight, especially when compared to calorie restriction or exercise interventions. These findings suggest that while yoga may not consistently reduce %BF across all populations, it can contribute to meaningful changes in body composition when practiced regularly and as part of a structured program [40,41]. Current guidelines suggest that even a modest reduction in %BF (<5%) is sufficient to produce clinically meaningful improvements in insulin sensitivity, lipid profiles, and systemic inflammation [42]. Systematic reviews and meta-analyses have shown that yoga interventions can significantly improve lipid profiles by reducing total cholesterol, LDL, triglycerides, and VLDL while increasing HDL. These lipid changes are important for reducing cardiovascular risk and improving metabolic health, supporting the potential of yoga to positively influence insulin sensitivity and systemic inflammation pathways relevant to metabolic syndrome [43,44]. Furthermore, the consistent adaptation observed across all age subgroups suggests that Bikram yoga may serve as a viable non-pharmacological intervention to combat sarcopenic obesity. This emerging chronic condition, common in aging women, is characterized by the simultaneous loss of muscle quality and increased adiposity, both of which significantly impair functional independence [45]. Yoga’s low-impact nature, combined with its capacity to enhance muscle strength, balance, and flexibility, makes it a promising intervention for managing sarcopenic obesity in aging populations. Reviews highlight yoga’s role in reducing cardiovascular risk factors and systemic inflammation through both physical activity and stress reduction mechanisms, which are critical for preserving functional independence among older adults [1,46,47].

The observed BF% reduction of 6.07% (*p* < 0.0001) exceeds the clinical threshold (>5%) for metabolic benefits. Although biochemical or metabolic markers were not directly assessed in this study, reductions in body fat are generally considered clinically relevant because they have been associated with improvements in insulin sensitivity, lipid profiles, and systemic inflammation in the previous literature. Therefore, the magnitude of %BF reduction observed here may be considered potentially meaningful from a public health perspective, although these mechanisms were not directly evaluated in the present study [25]. In age subgroups, consistent reductions (−5.39% to −6.62%) suggest efficacy across the female lifespan, particularly for postmenopausal women at risk of MetS. Bikram yoga’s heated environment and asana sequence may amplify fat metabolism and anti-inflammatory effects, as suggested by yoga trials among obese adults with MetS. Several studies suggest that yoga interventions may influence metabolic and inflammatory pathways, including adipokine regulation and insulin sensitivity. However, these mechanisms or physiological markers were collected. The current findings should therefore be interpreted as anthropometric evidence of changes in body composition rather than as direct proof of metabolic adaptation [24].

A significant aspect of this study was that it was not a generic physical activity but a structured protocol: each 90-minute Bikram yoga session was conducted in a heated room at approximately 40 °C and with controlled humidity, following the method’s original sequence. This thermal context, along with the specificity of the asanas (postures) performed, which include isometric components, stretching, balance, and respiratory control, likely contributed significantly to improvements in overall body composition in adult women.

It is important to emphasize that despite the lack of a control group, comparing values recorded at different time points (T0, T1, T2, T3) allowed a reliable assessment of the trend of change over time, thereby supporting experimental observation in a real-world context. Furthermore, the methodological consistency of the surveys (conducted by the same operator, using standardized instruments) ensures homogeneity in the collected data.

This study has limitations that should be acknowledged. First, the absence of a non-intervention control group prevents causal inference and limits the ability to attribute the observed changes exclusively to Bikram yoga practice. Natural lifestyle changes, seasonal effects, or unmonitored dietary modifications may have contributed to the observed reductions in %BF. Second, adherence to the training program was not formally quantified, and dietary intake was not monitored, both of which may confound body-composition studies. Third, the estimation of %BF in the present study relied on indirect anthropometric prediction equations rather than criterion methods such as DXA or hydrodensitometry [48]. Although the prediction models implemented in the Exercise Science Toolkit are widely cited and validated against criterion methods, each equation was initially developed on specific populations (e.g., sex, age, ethnicity, and training status). As such, some degree of bias and variability may be expected when applying them to different cohorts. Furthermore, the absolute accuracy of skinfold-based estimations is generally limited, with a standard error of estimate typically ranging from 3% to 5% body fat compared with reference techniques. For this reason, using multiple equations and reporting a standard range of predicted values, as performed in the present study, provides a more conservative and robust approach than reliance on a single formula [27]. Nonetheless, the results should be interpreted as indicative of relative differences and trends in body composition rather than as exact quantifications of absolute fat mass. Finally, the relatively small sample size and single-arm observational design limit generalizability.

Overall, the present findings provide evidence of longitudinal changes in %BF estimated from anthropometry. Any interpretation regarding metabolic health, chronic disease risk, or physiological mechanism should be considered hypothesis-generating and based on previous literature rather than direct measurements from this study.

These observations suggest that Bikram yoga is a comprehensive, intense, and beneficial practice, capable of producing positive effects on body composition in adult women after just a few weeks, with a greater impact on younger and/or more trained subjects, but still effective in older populations. The combination of physical activity, breath control, environmental conditions, and mental discipline makes this type of yoga a valid proposition for prevention and maintaining good health, in an era where psychophysical well-being and body weight management are increasingly central.

## 5. Conclusions

This experimental study suggests that Bikram yoga, when practiced regularly for 6 months, is a valuable tool for positively modifying body composition in a heterogeneous sample of adult women. A progressive reduction in %BF was observed across all age groups, becoming evident early in the intervention, supporting the importance of regular and sustained participation. These results suggest that Bikram yoga is a valid alternative to other forms of physical exercise, particularly for adult, less physically fit women seeking an effective yet sustainable long-term activity that allows for socialization. It is a valuable tool for active aging and social inclusion strategies. Thus, regular Bikram yoga practice may represent a feasible and engaging form of physical exercise for adult women.

Although this study represents a rare six-month longitudinal protocol on Bikram yoga in adult women, showing consistent effectiveness in %BF reduction over time, limitations include a small sample size, the lack of a control group, potential confounding by adherence/diet, and indirect %BF estimation. Future randomized controlled trials including dietary monitoring, adherence assessment, and metabolic biomarkers are needed to confirm and extend these findings. In conclusion, the study supports the idea that a structured, regular, and monitored practice of Bikram yoga can lead to measurable improvements in body composition in adult women, reinforcing the notion that this discipline can be considered not only a holistic practice, but also an intervention for promoting health and physical well-being.

## Figures and Tables

**Table 1:** Changes in %BF across time points in the overall sample and age subgroups. Values are presented as the mean ± standard deviation. Percentage change (%) indicates relative change from baseline (T0). The main effect of time was assessed using repeated-measures ANOVA, with effect size reported as partial eta squared (*η_p_*^2^). Pairwise comparisons between time points (T0–T1, T0–T2, T0–T3) show the %BF reduction between the specified assessments, and exact *p*-values correspond to Šidák adjusted post hoc tests.

Parameter	T0	T1	T2	T3	T0 vs. T1 (*p* Value)	T0 vs. T2 (*p* Value)	T0 vs. T3 (*p* Value)	*η_p_* ^2^
%BF	n = 22 29.45% ± 5.63%	n = 22 25.74% ± 5.41%	n = 22 24.65% ± 5.59%	n = 22 23.38% ± 5.20%	−3.17% (*p* = 0.000001)	−4.80% (*p* = 0.000001)	−6.07% (*p* = 0.000001)	0.924
%BF subgroup A (years 20–35)	n = 8 28.54% ± 5.75%	n = 8 24.83% ± 4.75%	n = 8 23.54% ± 4.96%	n = 8 21.92% ± 4.38%	−3.71% (*p* = 0.002055397)	−5% (*p* = 0.000162635)	−6.62% (*p* = 0.000050771)	0.911
%BF subgroup B (years 36–50)	n = 9 28.02% ± 5.70%	n = 9 24.29% ± 5.87%	n = 9 23.19% ± 6.05%	n = 9 22.06% ± 5.40%	−3.73% (*p* = 0.000014)	−4.83% (*p* = 0.000006)	−5.96% (*p* = 0.000002)	0.939
%BF subgroup C (years 51–65)	n = 5 33.48% ± 4.14%	n = 5 29.79% ± 4.31%	n = 5 29.06% ± 4.00%	n = 5 28.09% ± 3.76%	−3.69% (*p* = 0.001072)	−4.42% (*p* = 0.000918)	−5.39% (*p* = 0.001051)	0.960

## Data Availability

The data presented in this study are available on request from the corresponding author. Due to privacy and ethical considerations related to the anonymity of the human participants, the data cannot be made publicly available.
